# Effect of paraquat-induced oxidative stress on gene expression and aging of the filamentous ascomycete *Podospora anserina*

**DOI:** 10.15698/mic2014.07.155

**Published:** 2014-06-22

**Authors:** Matthias Wiemer, Heinz D. Osiewacz

**Affiliations:** 1 Institute of Molecular Biosciences and Cluster of Excellence Frankfurt Macromolecular Complexes; Department of Biosciences; J W Goethe University; Max-von-Laue-Str. 9, 60438 Frankfurt, Germany.

**Keywords:** Aging, paraquat, oxidative stress, transcriptome, copper, programmed cell death, mitochondria

## Abstract

Aging of biological systems is influenced by various factors, conditions and processes. Among others, processes allowing organisms to deal with various types of stress are of key importance. In particular, oxidative stress as the result of the generation of reactive oxygen species (ROS) at the mitochondrial respiratory chain and the accumulation of ROS-induced molecular damage has been strongly linked to aging. Here we view the impact of ROS from a different angle: their role in the control of gene expression. We report a genome-wide transcriptome analysis of the fungal aging model *Podospora anserina *grown on medium containing paraquat (PQ). This treatment leads to an increased cellular generation and release of H_2_O_2_, a reduced growth rate, and a decrease in lifespan. The combined challenge by PQ and copper has a synergistic negative effect on growth and lifespan. The data from the transcriptome analysis of the wild type cultivated under PQ-stress and their comparison to those of a longitudinal aging study as well as of a copper-uptake longevity mutant of *P. anserina* revealed that PQ-stress leads to the up-regulation of transcripts coding for components involved in mitochondrial remodeling. PQ also affects the expression of copper-regulated genes suggesting an increase of cytoplasmic copper levels as it has been demonstrated earlier to occur during aging of *P. anserina* and during senescence of human fibroblasts. This effect may result from the induction of the mitochondrial permeability transition pore via PQ-induced ROS, leading to programmed cell death as part of an evolutionary conserved mechanism involved in biological aging and lifespan control.

## INTRODUCTION

Aging of biological systems is a complex process which is characterized by irreversible functional impairments and ultimately leads to death of the system. The process is under the control of genetic, environmental and stochastic traits. According to the ‘mitochondrial free radical theory of aging’ (MFRTA), impairments are caused by molecular damage resulting from the activity of reactive oxygen species (ROS) generated as by-products during respiration 1. More recently, due to the accumulation of counter-intuitive, non-consistent or even contradictory data, the MFRTA has been challenged 2,3. It appears that the rather simplistic relationship of ROS generation and the age-related accumulation of bulk molecular damage 4,5,6 are not sufficient to explain aging and a more complex scenario is effective 6,7,8 which remains to be elucidated in more detail.

We use *Podospora anserina* as an experimentally accessible aging model to elucidate the mechanistic basis of organismal aging 9,10. This filamentous fungus is characterized by a short lifespan. Various pathways and processes including mitochondrial DNA instability 11, cellular copper homeostasis 12, respiration 13, ROS generation and scavenging 14, proteostasis 15,16, mitochondrial dynamics 17, autophagy 18,19, and apoptosis 20,21 have been shown to affect aging. A recent age-related, genome-wide transcriptome analysis of the *P. anserina* wild type revealed evidence for interactions between pathways leading to compensatory effects once a particular component and pathway is affected 19.

Apart from their damaging role, ROS are active in signaling and control of gene expression. This function may link genetic, environmental and stochastic processes involved in aging and lifespan control and can help to explain unexpected and counter-intuitive experimental data. After having investigated genome-wide transcriptome profiles of *P. anserina* wild-type cultures of different age and of a long-lived mutant in which mitochondrial ROS generation is reduced compared to the wild type 22,23, we now set out to analyze the impact of increased oxidative stress on global gene expression. Using paraquat (PQ) as a generator of the superoxide anion at the mitochondrial respiratory chain 24,25, the site at which this ROS is generated also during normal aging, we experimentally induced strong cellular oxidative stress in wild-type cultures of different age and found that this treatment has profound effects on gene expression, growth and lifespan.

## RESULTS AND DISCUSSION

### Dose-dependent induction of oxidative stress by PQ

**Figure 1:**
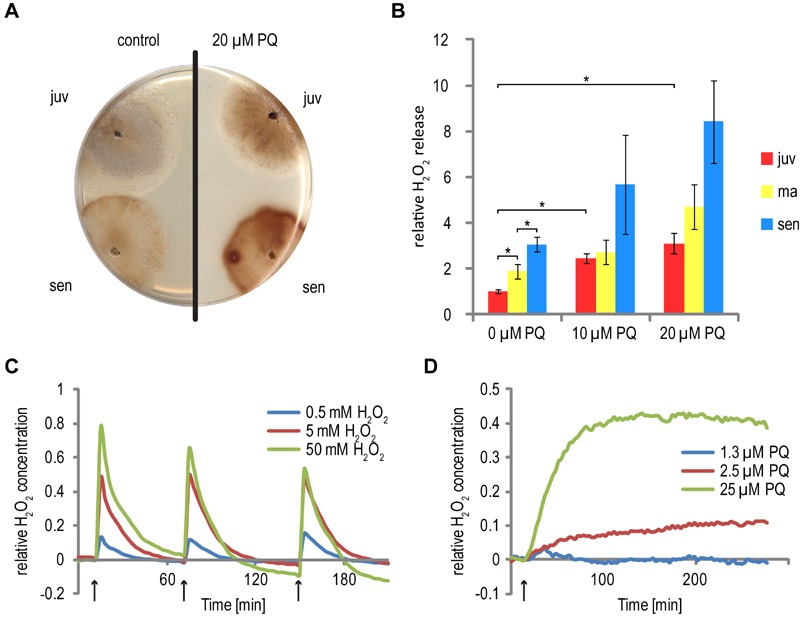
FIGURE 1: Increased hydrogen peroxide generation and release by *P. anserina* cultures during aging and PQ-stress. **(A)** After 2 days of ascospore germination, fungal mycelia were either directly challenged with 20 µM PQ (juv) or grown for 11 days on M2 medium to obtain a senescent stage. The cultures were subsequently incubated for 4 days on M2 with 20 µM PQ. As a control, both age stages (juv: 6 days; sen: 17 days) were cultivated on PQ-free M2 medium. After this time, DAB-staining-solution was poured on the cultures. The reaction with secreted H_2_O_2_ leads to the formation of a dark brown pigment. **(B)** Quantitative measurement of H_2_O_2_ release. *P. anserina* cultures were essentially grown as described in (A). Instead of on M2, cultures were grown on PASM medium until reaching a final age of 6 days (juv), 11 days (ma) and 15 days (sen), respectively. Concentrations of 0 µM, 10 µM and 20 µM PQ were administered. Bars indicate mean ± standard error of H_2_O_2_ release relative to juvenile untreated cultures. n = 16 - 41. Asterisks indicate pV < 0.01. **(C and D)** Relative changes in H_2_O_2_ concentration in the cytoplasm. *P. anserina* strains producing the redox sensitive HyPer protein, localized to the cytoplasm, were germinated for 2 days on germination medium and grown for 4 days on M2 agar plates. The mycelium was punched out of the agar, transferred to a 96 well plate and soaked in 60 µl water. Subsequently, H_2_O_2_ **(C)** or PQ **(D)** was added at the indicated times (arrows) to a final concentration of 0.5 mM, 5 mM and 50 mM or 1.3 µM, 2.5 µM and 25 µM, respectively. The fluorescence of the mycelium after exitation at 488 ± 5 nm (oxidized HyPer) and 420 ± 5 nm (reduced HyPer) was measured at 530 ± 20 nm. The graphs show the ratio of oxidized to reduced HyPer normalized to a untreated *HyPer* strain.

In a series of experiments, we investigated the response of the *P. anserina* wild type to PQ-stress. First, we determined the release of H_2_O_2_ by cultures of different age. These age-specific cultures were generated from mycelia that developed after germination of ascospores using pieces of these mycelia either directly (juvenile (juv) cultures) or after transfer to solid, PQ-free medium and incubation at 27°C for 5 days (middle-aged (ma) cultures) and 9-11 days (senescent (sen) cultures), respectively. From these cultures hyphal tips of the growth front were transferred to solid medium with different PQ concentrations and cultured for four additional days. After this period of time, the release of H_2_O_2_ was visualized as a dark brown pigment that forms on agar plates after reaction with a 3,3-diaminobenzidine (DAB) containing solution (Figure 1A). An increase of H_2_O_2_ release during aging and after PQ treatment was observed in particular at the growth front of senescent and of PQ-stressed cultures. For a quantitative and more detailed photometric analysis, we transferred cultures of defined age to 96-well plates, incubated them with DAB staining solution and determined the absorbance of the recovered solution (Figure 1B). In cultures of older age, a clear increase of H_2_O_2_ release is observed. Senescent cultures release approximately three times more H_2_O_2_ than juvenile cultures, verifying the known increase of oxidative stress during aging 17. Consistently, also in cultures of different age (6 days, 11 days, 15 days) grown on medium containing 10 µM and 20 µM PQ, respectively, a significant increase of H_2_O_2_ release is observed. The release is highest in senescent cultures grown on 20 µm PQ.

Assumingly, the degree of H_2_O_2_ release by *P. anserina* cultures is proportional to cellular ROS levels in the organism. In order to validate this assumption experimentally, we investigated *P. anserina* strains expressing the gene coding for the redox sensitive HyPer reporter protein 26. This protein is specifically oxidized by H_2_O_2_ and can be reverted to the reduced form by endogenous glutaredoxin. *P. anserina* strains expressing *HyPer* respond to the application of H_2_O_2_ by a change in fluorescence during excitation at 488 nm (oxidized HyPer) and 405 nm (reduced HyPer) in a dose-dependent manner. Repeated addition of H_2_O_2_ over time can be followed using this system (Figure 1C). In accordance with the observed dose-dependent increase of H_2_O_2_ release from cultures subjected to increasing PQ-stress, strains expressing *HyPer* on media containing different concentrations of PQ revealed a dose-dependent response of relative fluorescence ratios over time (Figure 1D). These data verify the conclusion that growth of *P. anserina* on PQ-containing medium indeed increases endogenous (cellular) H_2_O_2_ stress.

### Age-dependent effect of PQ-stress on global gene expression 

To analyze the impact of defined oxidative stress of *P. anserina* cultures of different age on gene expression and to compare this effect with existing data from a study of *P. anserina* cultures aged under standard growth conditions without PQ-stress 19, we treated cultures of the wild type of defined age (6 days, 11 days, 15 days) for 24 h with 200 µM PQ and subsequently isolated total RNA. In these experiments, we used a higher PQ concentration than the concentration in experiments with solid PASM medium. This was necessary, because we aimed to induce strong effects on gene expression by oxidative stress and to investigate direct effects of superoxide rather than secondary effects that occur during longer exposure. Moreover, the effect of PQ on growth is lower in complete medium used for the isolation of RNA (data not shown).

Using equal amounts of pooled RNA from three individual wild-type cultures, a SuperSAGE analysis was performed (GenXPro; Frankfurt, Germany). Between 14 and 20 million sequence tags were obtained for each sample, corresponding to about 10,000 of the 10,644 predicted *P. anserina* genes. Transcript counts of PQ-stressed cultures were compared to those of an existing data set from a previous study of cultures of similar age, grown on standard PQ-free medium. For the identification of genes with a robust differential expression, we set the relative expression threshold to > 3 and pV < 0.01.

**Figure 2:**
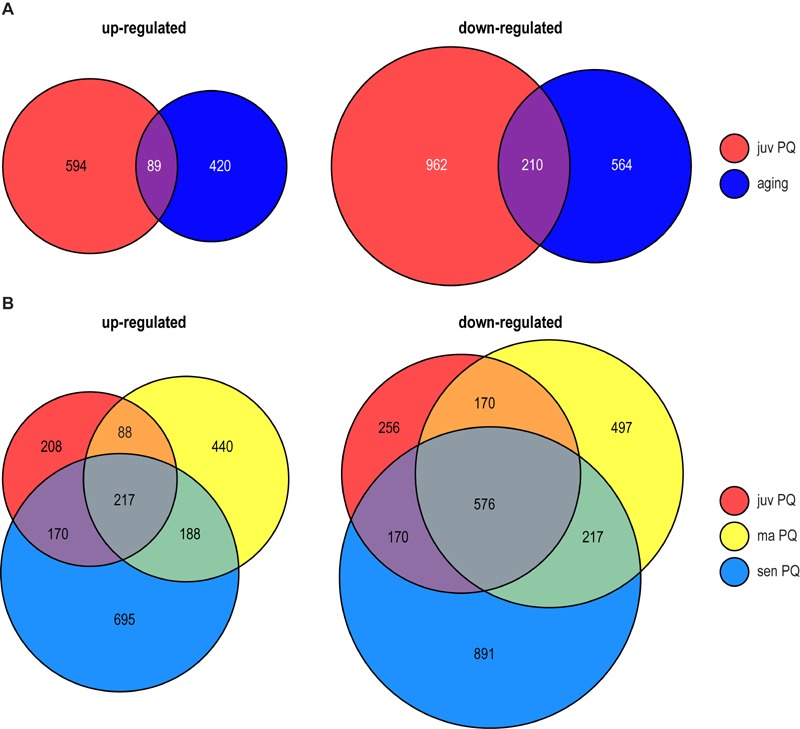
FIGURE 2: Differential transcript abundance during PQ-stress and aging. Venn diagrams of up- (left) and down- (right) regulated transcripts (factor 3; pV < 0.01) identified in transcriptome analysis. Factors are the quotient of tpm (tags per million) of treated (200 µM PQ for 24 h or aging) and control samples. **(A)** Venn diagram of transcripts regulated in juvenile PQ-treated (juv PQ = tpm 6 days PQ / tpm 6 days control, red) and aged cultures (tpm 14 days control/ tpm 6 days control, dark blue) and **(B)** juvenile PQ-treated (juv PQ = tpm 6 days PQ /tpm 6 days control, red), middle-aged PQ-treated (ma PQ = tpm 11 days PQ / tpm 11 days control, yellow) and senescent PQ-treated (sen PQ = 15 days PQ/ 14 days control, light blue) *P. anserina*. The size of a circle represents the number of regulated transcripts.

Compared to juvenile (6 days) cultures grown on PQ-free medium, 683 (594 plus 89) genes were found to be up-regulated in PQ-stressed cultures of the same age (Figure 2A; Table 1). In comparison, 509 (420 plus 89) transcripts were up-regulated in senescent cultures aged on standard growth medium. A comparison of the up-regulated gene sets revealed that aging and PQ-stress has a similar effect on 89 transcripts (Figure 2A; Table 1). This number is significantly higher than the number of 35 which is statistically expected if regulation would be random and independent of ROS (Table 1). Since the experimentally administered PQ-stress is greater and more rigorous than it occurs during normal aging, where oxidative stress continuously increases over a longer period of time 27,28, more genes (683) are induced in the PQ-stressed than in the ‘aging’ (509) group.

**Table 1:** Global gene regulation in the *P. anserina* wild type stressed by PQ and during aging. The table shows the number of differentially regulated genes (factor < 3; pV < 0.01) of all analyzed transcriptome data. In the individual columns two data sets are compared (i.e. aging and PQ stress in juvenile cultures), respectively. The lines show the number of differentially expressed genes at the specified treatment. The amount of differentially regulated transcripts in the specific direction in both treatments is depicted in the line ‘conjoint regulated’. The statistical expectation (‘stat. expectation’) provides the number of genes which is expected to be regulated if the two treatments initiate random gene regulation. The p-value was calculated by Fisher’s exact test. Since the calculation of p-values lower than 2.2e-16 are identical, we included the odds ratio to clarify the level of similarity in transcript generation. The odds ratio is a measure for statistical dependence. A high odds ratio indicates a high probability for a regulation of a gene in the same direction during both compared condition.

	**Aging / PQ juv**		**PQ juv / PQ ma**		**PQ juv / PQ sen**		**PQ ma / PQ sen**		**PQ juv / Grisea**	
	**up**	**down**	**up**	**down**	**up**	**down**	**up**	**down**	**up**	**down**
Aging	509	774								
PQ juv	683	1172	683	1172	683	1172			683	1172
PQ ma			933	1460			933	1460		
PQ sen					1270	1854	1270	1854		
Grisea									608	556
Conjoint regulated	89	210	305	746	387	746	405	793	167	267
Stat. expectation	35	90	64	172	87	217	119	273	43	67
p-value	2.2e-16	2.2e-16	2.2e-16	2.2e-16	2.2e-16	2.2e-16	2.2e-16	2.2e-16	2.2e-16	2.2e-16
Odds ratio	3.19	3.21	11.06	19.71	12.51	12.23	7.20	8.30	6.29	8.41

To further investigate the correlation of transcript regulation in different transcriptome data, Fisher’s exact test was applied to give a measure of similarity of the compared transcriptome data. Since the investigated data sets revealed identical, low p-values of < 2.2e-16, we calculated odds ratio provided by Fisher’s exact test as a measure of similarity of two data sets. For instance, the comparison of up-regulated transcripts from cultures aged on PQ-free growth medium and PQ-stressed juvenile cultures revealed an odds ratio of 3.19 (Table 1). This indicates a 3.19 times higher probability of transcripts of one group (aging) to be also up-regulated in the other group (PQ-stressed). Overall, both statistical tests revealed for all compared sets of data a significant number of genes that are similarly regulated under the investigated conditions (e.g., PQ-stress, aging).

The number of genes which is down-regulated during PQ-stress of juvenile cultures (1,172) and during aging (774) is higher than the number of up-regulated genes (683, 509) (Figure 2A). We found 210 genes being down-regulated during both PQ-stress and normal aging. Again, this number is significantly higher than the 90 transcripts statistically expected if regulation would be random (Table 1). As indicated by similar odd rations (Table 1; odds ratio of 3.21 and 3.12, respectively) the proportion of similarly regulated genes is comparable in both, the up- and down regulated gene fraction.

During normal aging, oxidative stress increases continuously and individuals of different age have to deal with different levels of ROS (Figure 1). This situation may affect the ability to respond to exogenous PQ-stress in different directions. On the one hand, the age-related ROS accumulation could enhance survival during subsequent ROS treatment as a result of an adaptation to mild stress, a process called hormesis 29,30,31. On the other hand, general fitness differs in cultures of different age. Cultures of older age may therefore be less effective in dealing with additional stress.

In order to test the effect of aging on the ability to deal with exogenous PQ-stress, we compared the transcriptome data of *P. anserina* cultures of three different ages (juv, ma, sen) treated for 24 h with 200 µM PQ. The data revealed 217 up-regulated and 576 down-regulated transcripts, respectively, in all three age stages after applying PQ-stress (Figure 2B). These are far more genes than the corresponding statistically expected number of 8 and 31 randomly regulated genes, indicating a controlled PQ-dependent regulation of these genes, which is independent of age. Moreover, the comparison of the transcriptomes of cultures of different age (juv, ma, sen) treated with PQ revealed that the number of differentially expressed genes increases with the age of the investigated culture. We found 683, 933, and 1,270 transcripts up- and 1,172, 1,460, and 1,854 transcripts down-regulated in juvenile, middle-aged and senescent cultures, respectively (Figure 2B), indicating an age-dependent ROS regulation of gene expression with the strongest effect in strains of the oldest age, which also have the highest ROS levels (Figure 1B).

### Gene Ontology enrichment analysis 

In order to evaluate which molecular pathways respond to PQ-stress, we performed a Gene Ontology (GO) enrichment analysis of PQ-stress regulated genes in juvenile, 6 days old *P. anserina* cultures. Strikingly, genes encoding proteins of the ‘mitochondrial matrix’, the ‘mitochondrial inner membrane’ and the ‘mitochondrial intermembrane space’ as well as other categories related to mitochondria are enriched in the group of 683 genes up-regulated 3-fold or higher by PQ-stress (Table 2). It is possible that proteins in the vicinity of the ROS-generating electron transport chain are preferentially damaged during PQ-stress 32. Up-regulation of these genes could compensate for damaged and degraded proteins. Concordantly, transcripts of genes coding for proteins involved in this kind of mitochondrial remodeling, including those active in transport of components into mitochondria, the organization of mitochondria, mitochondrial translation, ubiquinone biosynthesis and mitochondrial genome maintenance are enriched in the group of up-regulated genes (Table 2).

**Table 2:** GO enrichment analysis of transcriptome data of juvenile PQ-stressed *P. anserina *showing differential expression. All differentially expressed genes (Factor < 3; pV < 0.01) were analyzed. GO terms with pV < 1E-5 are shown. The GO terms referred to in the text are highlighted in bold. CC: Cellular Component; BP: Biological Process; C: Count (number of genes of respective GO Term in the group (up- or down-regulated)); S: Size (total number of *P. anserina* genes with the respective GO term).

**Up-regulated**					
	**GO ID **	**p-value **	**C**	**S**	**Description**
CC	GO:0044429	4.08E-22	101	581	mitochondrial part
	GO:0005739	8.68E-19	144	1121	mitochondrion
	GO:0005743	5.39E-17	57	261	**mitochondrial inner membrane**
	GO:0019866	2.34E-16	57	269	organelle inner membrane
	GO:0005740	2.15E-15	71	408	mitochondrial envelope
	GO:0031966	2.06E-13	65	386	mitochondrial membrane
	GO:0044455	6.74E-13	38	159	mitochondrial membrane part
	GO:0005759	7.56E-12	43	212	**mitochondrial matrix **
	GO:0031967	2.26E-09	75	584	organelle envelope
	GO:0031975	4.99E-09	77	617	envelope
	GO:0005744	2.86E-07	8	13	mitochondrial inner membrane presequence translocase complex
	GO:0005758	5.06E-06	12	40	**mitochondrial intermembrane space **
	GO:0031305	4.79E-05	6	12	integral to mitochondrial inner membrane
BP	GO:0006839	1.97E-14	34	113	mitochondrial transport
	GO:0007005	2.31E-13	50	243	mitochondrion organization
	GO:0006626	1.94E-09	19	57	protein targeting to mitochondrion
	GO:0070585	3.75E-09	19	59	protein localization to mitochondrion
	GO:0009060	1.40E-07	25	117	aerobic respiration
	GO:0030150	7.04E-07	9	18	protein import into mitochondrial matrix
	GO:0006744	1.61E-06	8	15	ubiquinone biosynthetic process
	GO:0045333	4.81E-06	33	215	cellular respiration
	GO:0065002	5.37E-06	11	33	intracellular protein transmembrane transport
	GO:0055114	8.04E-06	49	388	oxidation-reduction process
	GO:0032543	9.53E-06	15	62	mitochondrial translation
	GO:0017038	1.16E-05	22	120	protein import
	GO:0015980	2.10E-05	36	261	energy derivation by oxidation of organic compounds
	GO:0042180	2.10E-05	81	781	cellular ketone metabolic process
	GO:0007007	3.87E-05	7	16	inner mitochondrial membrane organization
	GO:0006091	3.89E-05	43	344	generation of precursor metabolites and energy
	GO:0044281	4.66E-05	130	1438	small molecule metabolic process
	GO:0006520	8.47E-05	59	541	cellular amino acid metabolic process
	GO:0000002	8.66E-05	11	43	mitochondrial genome maintenance
	GO:0006627	9.41E-05	4	5	protein processing involved in protein targeting to mitochondrion
MF	GO:0008320	7.45E-08	11	23	protein transmembrane transporter activity
	GO:0015450	3.15E-07	10	21	P-P-bond-hydrolysis-driven protein transmembrane transporter activity
	GO:0016491	8.58E-05	166	1920	oxidoreductase activity
**Down-regulated**					
	**GO ID **	**p-value**	**C **	**S**	**Description**
BP	GO:0042438	4.97E-07	9	21	**melanin biosynthetic process**
	GO:0032787	6.47E-07	32	236	monocarboxylic acid metabolic process
	GO:0006725	4.29E-06	28	209	cellular aromatic compound metabolic process
	GO:0006582	5.96E-06	9	27	melanin metabolic process
	GO:0044550	8.58E-06	10	35	secondary metabolite biosynthetic process
	GO:0019748	1.57E-05	11	45	secondary metabolic process
	GO:0006083	7.08E-05	6	15	acetate metabolic process
MF	GO:0016491	8.73E-17	193	1920	**oxidoreductase activity **
	GO:0004497	5.16E-11	49	298	** monooxygenase activity **
	GO:0004312	2.80E-07	16	60	**fatty acid synthase activity **
	GO:0016705	2.82E-07	54	442	oxidoreductase activity, acting on paired donors, with incorporation or reduction of …
	GO:0005506	3.36E-07	58	492	iron ion binding
	GO:0016701	1.99E-06	23	129	oxidoreductase activity, acting on single donors with incorporation of molecular oxygen
	GO:0020037	2.54E-06	45	366	heme binding
	GO:0046906	2.95E-06	45	368	tetrapyrrole binding
	GO:0046872	7.29E-06	201	2627	metal ion binding
	GO:0004316	7.77E-06	10	31	3-oxoacyl-[acyl-carrier-protein] reductase (NADPH) activity
	GO:0046914	8.28E-06	162	2022	transition metal ion binding
	GO:0009055	2.14E-05	46	409	electron carrier activity
	GO:0043169	2.32E-05	208	2781	cation binding
	GO:0016614	3.58E-05	51	480	oxidoreductase activity, acting on CH-OH group of donors
	GO:0070330	4.41E-05	10	37	aromatase activity
	GO:0016616	5.28E-05	44	399	oxidoreductase activity, acting on the CH-OH group of donors, NAD or NADP as acceptor
	GO:0016702	6.01E-05	19	117	oxidoreductase activity, acting on single donors with incorporation of molecular oxygen…
	GO:0008709	7.86E-05	5	9	cholate 7-alpha-dehydrogenase activity

**Figure 3:**
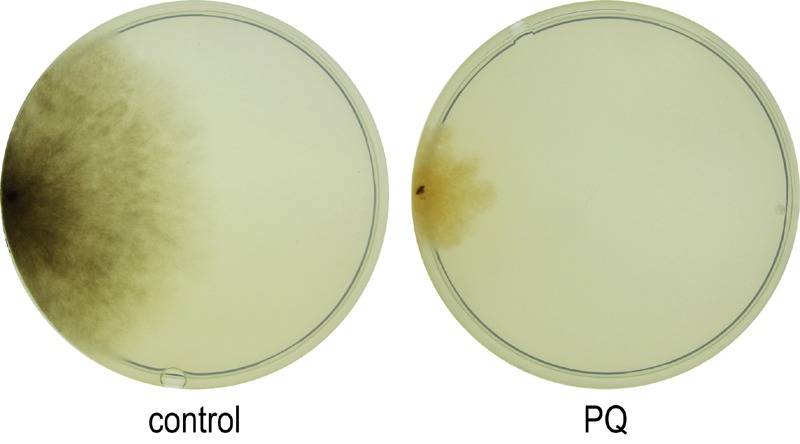
FIGURE 3: Effect of PQ-stress on the phenotype of *P. anserina*. Cultures of the wild type grown for 8 days on PQ-free CM medium or on CM medium supplemented with 200 µM PQ.

Among the 1,172 genes which are down-regulated at least 3-fold after applying PQ-stress to juvenile cultures (Figure 2A, right), no enrichment of transcripts coding for proteins located in a specific cellular compartment is found. Instead, GO terms for several biological processes are enriched (Table 2). The down-regulation of genes coding for components of ‘melanin biosynthetic processes’ and ‘secondary metabolite biosynthetic process’ is in concordance with the decreased pigmentation of cultures grown under PQ-stress (Figure 3). Both processes are energy consuming and therefore may be down-regulated. After applying PQ-stress, the genes Pa_2_510, Pa_5_11880, Pa_7_11610, Pa_5_1990 coding for products involved in the synthesis of melanin 33 are down-regulated by factors between 2 and 560 (Figure S1A). It appears that, although melanin is known to protect against oxidative stress 34, under the investigated strong stress conditions this protective pathway is inactive. However, a strikingly similar regulation of these transcripts in the copper-depleted grisea mutant points towards a copper-dependent regulation of these genes, which will later be further discussed.

Among the most enriched molecular functions in the group of down-regulated genes are the GO terms ‘fatty acid synthase activity’, ‘oxidoreductase activity’ and ‘monooxygenase activity’ (Table 2). The synthesis of fatty acids is also an energy consuming process. Similar to our data, genes involved in fatty acid metabolism are down-regulated in PQ-stressed *Drosophila melanogaster* but not in aged flies 35. The GO term ‘oxidoreductase activity’ is among the enriched terms in both, up- and down-regulated transcripts. Referring to the H_2_O_2_-release (Figure 1), the generation of superoxide can be expected to have a strong effect on the redox status of the cell, which in turn seems to have an effect on regulation of enzymes with redox activity.

Next, we compared GO term enrichments of the current study with those of a previous analysis in which individuals were aged under standard growth conditions 19. One possibility leading to the same GO terms in the up- or down-regulated fraction of genes with a comparable p-value in our GO-term enrichment analysis is a similar gene regulation during aging and PQ-stress. If this is true, this would support a ROS-dependent regulation during aging. Such similarities can be observed for the up-regulation of genes with the GO term ‘mitochondrial part’ (GO: 0044429) and for the regulation of transcripts coding for proteins of the respiratory chain (GO:0005746; GO:0070469; GO:0045333) (Table 3). Transcripts coding for the proteasome and its assembly (GO:0000502; GO:0031597; GO:0043248) and for ribosomes (GO:0022626; GO:0044391; GO:0005840; GO0015935; GO:0022625; GO:0015934) are enriched in the down-regulated groups during both, aging and PQ-stress, except for the GO term ‘ribosomal subunit’ (GO:0044391), which refers to mitochondrial ribosomes and is up-regulated during PQ-stress.

**Table 3:** Comparison of enriched GO terms in the data set of PQ-stressed *P. anserina* cultures with those from an earlier longitudinal aging study 19. Terms that are enriched in the same group (up- or down-regulated) during aging and PQ-stress are highlighted in bold. Similar GO terms are grouped. CC: Cellular Component; BP: Biological Process; C: Count (number of genes of respective GO Term in the group (up- or down-regulated)); S: Size (total number of *P. anserina* genes with the respective GO term).

**Aging**						**juv PQ up**		**juv PQ down**	
**GO term**	**p-value**	**C**	**S**	**Term**	**direction**	**p-value**	**C**	**p-value**	**C**
44429	7.5E-03	152	581	**mitochondrial part**	up	1.03E-63	318		
44429	9.3E-06	171	581	mitochondrial part	down				
5746	5.2E-03	23	62	mitochondrial respiratory chain	down				
5746	2.1E-03	24	62	**mitochondrial respiratory chain**	up	9.08E-18	47		
70469	3.9E-03	27	75	**respiratory chain**	up	7.27E-16	51		
45333	2.4E-03	65	215	**cellular respiration**	up	1.09E-22	117		
45333	4.1E-04	68	215	cellular respiration	down				
502	1.7E-04	35	89	**proteasome complex**	down			5.75E-03	28
31597	1.6E-03	13	26	**cytosolic proteasome complex**	down			2.08E-03	12
34515	1.6E-03	13	26	**proteasome storage granule**	down			2.08E-03	12
43248	1.3E-03	12	23	**proteasome assembly**	down			7.83E-03	10
22626	3.5E-11	50	94	**cytosolic ribosome**	down			3.75E-11	47
5840	2.2E-08	126	367	**ribosome**	down			8.66E-04	97
22627	6.7E-07	23	39	**cytosolic small ribosomal subunit**	down			2.25E-06	21
15935	6.9E-07	30	58	**small ribosomal subunit**	down			1.00E-03	22
22625	2.9E-05	23	46	**cytosolic large ribosomal subunit**	down			9.10E-07	24
15934	4.2E-05	35	84	**large ribosomal subunit**	down			4.44E-04	30
44391	3.1E-09	61	137	ribosomal subunit	down	1.47E-09	65		
6090	2.2E-05	18	32	pyruvate metabolic process	down				

During aging of *P. anserina* we previously reported an enrichment of autophagy genes in a group of continuously up-regulated genes 19. In the current study, we did not find such an increase in abundance of autophagy transcripts during PQ-stress of juvenile *P. anserina*. In contrast, PQ-stress applied on senescent cultures resulted in the enrichment of GO terms of the categories ‘positive regulation of autophagy’ (GO: 0010508) and ‘regulation of autophagy’ (GO: 0010506) in the group of up-regulated genes (Table S1). These results seem to indicate that a regulation of the corresponding genes is ROS-dependent but requires additional factors and/or conditions as they accumulate in senescent cultures.

### ROS scavenging pathways

**Figure 4:**
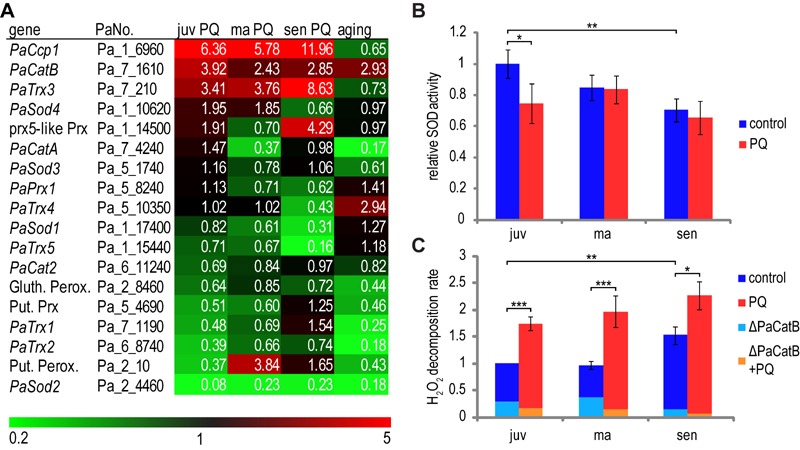
FIGURE 4: Effect of PQ-stress and aging on ROS-scavenging pathways. **(A) **Transcriptome data of ROS scavenger enzymes. Colors and numbers indicate relative expression of transcripts of the indicated genes and treatment. Relative expression was calculated by dividing tpm of juvenile PQ-stressed samples by tpm of juvenile control (juv PQ), middle-aged PQ-stressed samples by middle-aged control (ma PQ), senescent PQ-stressed samples by senescent control (sen PQ) and senescent control by juvenile control (aging). **(B,C)** The overall SOD activity and H_2_O_2_ decomposition rate were measured in protein extracts of juvenile (juv), middle-aged (ma) and senescent (sen) *P. anserina *cultures grown in PQ-free medium or in medium supplemented with 200 µM PQ. **(B)** Overall SOD activity is shown as mean relative expression to the juvenile wild type ± standard error. n = 6. Student’s t test p-values: juv to juv PQ pV = 0.049; juv to sen pV = 0.007. **(C)** Besides in wild-type strains (control and PQ), measurement of H_2_O_2_ decomposition was carried out in a Δ*PaCatB* strain to estimate the portion of activity related to PaCATB. Data of wild type is shown as mean ± standard error. n = 9 - 11 for wt; n = 1 for Δ*PaCatB*. Student’s t-test p-values of wild type: juv to sen pV = 0.004; juv to juv PQ pV = 7.05E-5; ma to ma PQ pV = 2.41E-5; sen to sen PQ pV = 0.016.

In *P. anserina* different pathways affecting oxidative stress are known to affect aging and longevity. Among these, ROS scavenging pathways are effective via enzymatic detoxification. In the genome of *P. anserina* a number of ROS scavenging enzymes is encoded. We investigated whether the corresponding genes are transcriptionally regulated after applying PQ-stress and during aging of cultures without PQ-stress. As indicated in Figure 4A, transcripts of *PaCcp1*, *PaCatB*, and *PaTrx3 *are up-regulated after applying PQ-stress. *PaCcp1*, the gene coding for cytochrome *c* peroxidase, which is located in the mitochondrial inter-membrane space, catalyzes the detoxification of H_2_O_2_ to water by oxidation of ferrocytochrome *c*. After application of PQ-stress, transcript levels increase by factors of 6.36 to 11.96 in juvenile and senescent cultures (Figure 4A). In contrast, decreased levels of this transcript are found during aging when juvenile and old cultures are compared. The only transcript that is up-regulated during aging and after PQ-stress is the transcript of *PaCatB* coding for a *P. anserina* catalase. Among the down-regulated transcripts, the *PaSod2* transcript is most effected during both, aging and PQ-stress. The gene encoding the Cu/Zn-SOD (PaSOD1) is only slightly up-regulated during aging. Administration of PQ-stress leads to a reduction of *PaSod1* transcript levels. This reduction increases with the age of the investigated culture.

To test whether the identified up- and down-regulation of *PaSod1* and *PaCatB* contributes to SOD and CAT-activities, respectively, we determined the overall SOD activity and the H_2_O_2_ decomposition activity of different *P. anserina* strains. SOD activity is significantly reduced during aging (Figure 4B). The observed decrease in juvenile cultures challenged with PQ-stress is consistent with the transcript data for *PaSod1* and *PaSod2*. This is not the case when middle-aged and senescent cultures are analyzed. In these age stages SOD activity is unchanged, although the transcripts of *PaSod1* and *PaSod2* are less abundant, indicating that there are other factors affecting SOD activity in these age stages.

Catalase activity in cell extracts of the wild type and the *PaCatB* deletion strain 36 was investigated *in vitro* by photometric determination of H_2_O_2_ decomposition. The analysis of the *PaCatB* deletion strain verifies that the major activity measured with this method, under the tested conditions, is PaCATB activity (Figure 4C). In the wild type, the increase of *PaCatB* transcripts during aging and PQ-stress observed in transcriptome data (Figure 4A) is reflected by increased PaCATB activity (Figure 4C), indicating a transcriptional regulation of this protein. It appears that *PaCatB* is induced by superoxide during aging and oxidative stress.

Carotenoids are organic pigments with antioxidant activity 37. Previously we have shown that a high level of β-carotene and other carotenoids increases the lifespan of *P. anserina *38. PaAL-1 and PaAL-2 are the rate limiting enzymes of the carotenoid biosynthesis. We found that, at least in juvenile cultures, PQ-stress leads to a clear increase in transcript levels by factors 6.15 and 5.88, respectively (Figure S1B). Also, PaAL-3 levels, coding for another component of the carotenoid biosynthesis pathway is increased after PQ-stress of juvenile *P. anserina* by a factor of 2.04. In older cultures the increase in transcript levels is not that clear. For *PaAl-2* and *PaAl-3* transcript levels do even decrease after PQ-stress. It appears that, at least in earlier stages of the life cycle, carotenoid biosynthesis is induced to counteract oxidative stress.

### PQ-stress induces a strong response on copper and iron metabolism

In previous work, a strong link between oxidative stress, aging and a stringent control of copper homeostasis has been established in *P. anserina* 12,14,22,39,40,41. This link was unraveled by the analysis of wild-type strains of different age and of the long-lived grisea mutant. During aging of the wild type, transcript levels of the copper-regulated genes become altered. While transcripts coding for the *P. anserina* metallothionein (PaMT1) are increased, those coding for a high affinity copper transporter (PaCTR3) and for PaSOD2 are reduced. Consistently, PaSOD2 activity is reduced in old cultures 12,42. In addition, PaSOD1 activity, which is depending on the availability of cytoplasmic copper, is higher in wild-type strains of old age 12. Copper measurement by total reflection X-ray spectroscopy delineated the reason for these changes as an age-related increase of cytoplasmic copper levels 43. Copper-regulated transcription was demonstrated to be controlled by the copper-sensing transcription factor GRISEA. In senescent cultures, due to high cytoplasmic copper levels, GRISEA is inactivated and thus the expression of target genes is blocked. The same holds true in a *Grisea* loss-of-function mutant. Since transcription of *PaCtr3*, coding for a high affinity copper transporter, is dependent on the availability and activity of GRISEA, both in senescent wild-type strains as well as in the grisea mutant, copper-uptake is only possible via low affinity import. As a consequence, a switch from a copper-dependent standard to an iron-dependent alternative respiration, a strongly reduced generation of the mitochondrial superoxide anion and pronounced changes in gene expression are observed in the mutant 22,23,41.

**Figure 5:**
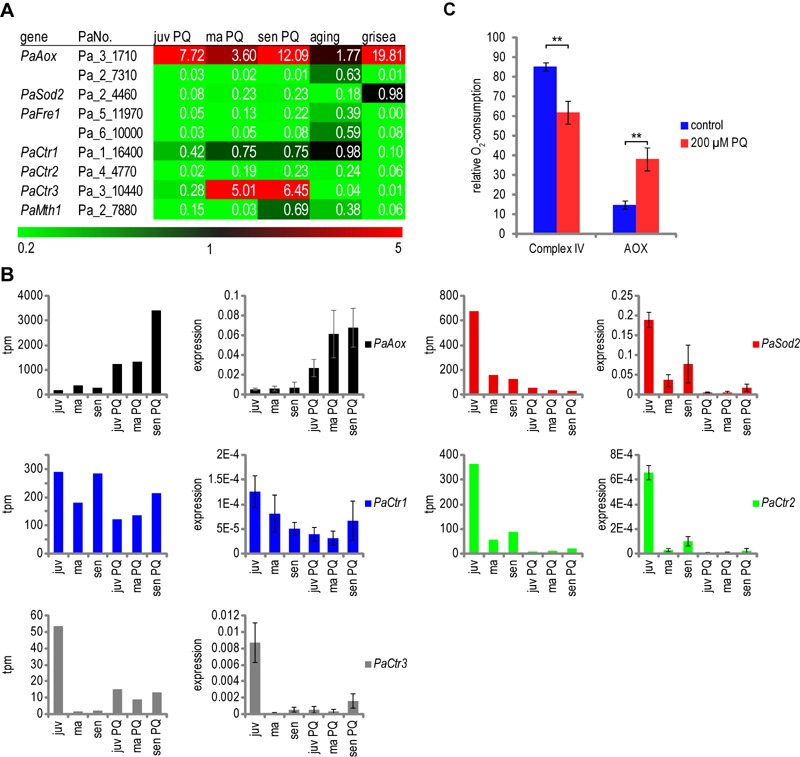
FIGURE 5: Effect of PQ-stress on the regulation of copper controlled genes. **(A) **Transcriptome data of copper and GRISEA regulated proteins. Colors and numbers indicate relative expression of transcripts of the indicated genes. Relative expression was calculated by dividing tpm of juvenile PQ-stressed samples by tpm of juvenile control (juv PQ), middle-aged PQ-stressed samples by middle-aged control (ma PQ), senescent PQ-stressed samples by senescent control (sen PQ), senescent control by juvenile control (aging) and grisea by wild-type control (grisea). **(B)** Verification of transcriptome data of the selected genes *PaAox*, *PaSod2*, *PaCtr1*, *PaCtr2*, and *PaCtr3*. Results from SuperSAGE analysis are shown in tpm (tags per million). The results of the corresponding qRT-PCR are depicted as relative expression levels to the reference genes *PaPorin* and *PaRpl19*. Data are displayed as mean ± standard error. n = 3. **(C)** Measurement of complex IV and PaAOX activity in control and PQ-stressed mycelia. Juvenile *P. anserina* was grown as described for transcriptome analysis. The oxygen consumption of 2 to 9 mg mycelium was measured in a high resolution respirometer. To determine the percentage of complex IV and PaAOX dependent respiration, a final concentration of 4 mM SHAM and 1 mM KCN was used to inhibit PaAOX and complex IV, respectively. Bars are mean ± standard error of 6 - 7 experiments with two different cultures. n = 20. Student’s t-test: pV = 0.0019.

The current analyses, in which we aimed to exaggerate superoxide stress via the application of PQ, indeed revealed a strong response of PQ-stress on gene expression linked to copper homeostasis. Genes either regulated by copper via yet unidentified transcription factors, or via the activity of GRISEA turned out to be down-regulated as occurs during aging or in the GRISEA deficiency mutant (Figure 5A). One exception is the regulation of *PaCtr3*, which at first glance seems to be regulated in middle-aged and senescent cultures stressed with PQ in the opposite direction as during wild type aging and in the grisea mutant. However, while the transcript numbers in the different samples, including the *PaCtr3 *mRNA in the untreated juvenile wild-type strain was in the range of 50 to 3,500 transcripts per million (tpm), transcript levels of *PaCtr3* in middle-aged untreated wild-type strains dramatically dropped to about 1 to 2 tpm. As demonstrated earlier, this is the result of increases in cytoplasmic copper leading to the repression of GRISEA activity 43. Because of this low expression of *PaCtr3*, we used qRT-PCR to verify the transcriptome data of this gene and of several other genes involved in copper homeostasis (Figure 5B). In order to obtain more accurate results, we used the two reference genes* PaPorin*, coding for a mitochondrial outer membrane protein and *PaRpl19*, coding for a subunit of the 60S ribosome.* PaPorin* is an established reference gene for qRT-PCR in *P. anserina*. Since the transcript abundance was found to vary during PQ-stress (307 to 1,307 tpm), we additionally included *PaRpl19*, which is stably and highly (1,031 to 1,183 tpm) expressed during aging and PQ-stress. The expression profiles of the investigated genes *PaAox*, *PaSod2*, *PaCtr1* and *PaCtr2* were basically verified by qRT-PCR experiments (Figure 5B). The down-regulation of *PaCtr3* transcripts during aging, as found in the Super-SAGE analysis, was also verified by qRT-PCR. However, the up-regulation of *PaCtr3 *by PQ-stress in middle-aged and senescent cultures is weaker in qRT-PCR than in the transcriptome data. The overall transcript abundance of *PaCtr3* in aged hyphae is probably so small that minor fluctuations in transcripts levels lead to strong effects on the relative transcript abundance, which however appear not to be of biological relevance. We assume that *PaCtr3* is only expressed in juvenile cultures in significant amounts and that transcription of this gene is silenced during aging and by PQ treatment. Taken together, the data reveal a PQ-induced down-regulation of genes involved in the control of cellular copper homeostasis.

This conclusion is confirmed by an up-regulation of transcripts coding for the alternative oxidase *PaAox* during PQ-stress. In order to verify that this up-regulation is reflected by a switch from a copper-dependent respiration via cytochrome *c* oxidase to an alternative respiration, as it is found in the copper-depletion mutant grisea, we determined the respiratory activity of complex IV and of PaAOX. Juvenile *P. anserina* were grown similar to the strains investigated in the transcriptome analysis. Oxygen consumption by complex IV or PaAOX was measured after inhibition of one of the two complexes revealing a significant reduction in cytochrome *c*-dependent respiration and an increase of AOX-dependent respiration from 15% to 39% in PQ-stressed strains (Figure 5C). PaAOX utilizes iron as cofactor. Iron-uptake in fungi takes place via two basic pathways 44. The first one, the reductive iron assimilation (RIA) pathway, is depending on the reduction of ferric iron to ferrous iron via the FRE1 reductase 45. Strikingly, during PQ-stress, aging, and in the grisea mutant, transcript level of the gene coding for the best *P. anserina* homolog of FRE1 (Pa_5_11970) is strongly reduced (Figure S1C). In yeast, uptake of Fe^2+^ is controlled by the iron transporter ScFTR1 and the copper oxidase ScFET3. The *ScFtr1* homolog Pa_6_4210 is not consistently regulated during PQ-stress. The three best *P. anserina* homologs of ScFET3 are encoded by Pa_6_4220, Pa_6_2250 and Pa_2_530. They are down-regulated during PQ-stress by factors 0.12 - 0.44 (Figure S1C) suggesting that RIA is impaired after PQ-stress. Under these conditions, like concluded for the grisea mutant, another iron-uptake system appears to be active. This pathway utilizes small iron binding proteins, termed siderophores. These molecules are secreted from the mycelium by a special siderophore transporter to bind extracellular iron and are subsequently transported back into the fungus and degraded to release iron for cellular use 46. Putative homologs to this iron transport system in *Aspergillus spec* have been identified in *P. anserina *by *in silico* analysis 23. The *Aspergillus fumigatus* proteins AfSIDA, AfSIDF and AfSIDD are necessary to produce the siderophore fusarin C. Genes of *P. anserina* coding for proteins with the highest degree of homology to these proteins (Pa_4_4430, Pa_5_4760 and Pa_3_11200) are up-regulated by factors between 2.04 and 5.09 during PQ-stress (Figure S1D). In *Aspergillus* the gene *AfSidC* is additionally needed for the synthesis of the intracellular siderophore ferricrocin. The gene coding for the best *P. anserina* homolog of *AfSidC*, Pa_4_4440, is up-regulated by factor 1.66 to 2.11 during PQ-stress. However, there are additional homologs with very high identity to AfSIDC. Furthermore, the genes coding for the putative homologs of the *A. nidulans* siderophore transporters MirB and MirC are mostly up-regulated during PQ-stress (Figure S2D). Overall, consistent with the observed impairment of copper-uptake, iron-uptake via siderophores appears to be induced during PQ-stress in *P. anserina*, although one has to keep in mind that this conclusion is only based on the homology to *Aspergillus* secondary metabolism genes. An increased iron influx would promote alternative respiration via iron dependant AOX in stressed cells with impairment in standard cytochrome *c*-dependent respiration.

**Figure 6:**
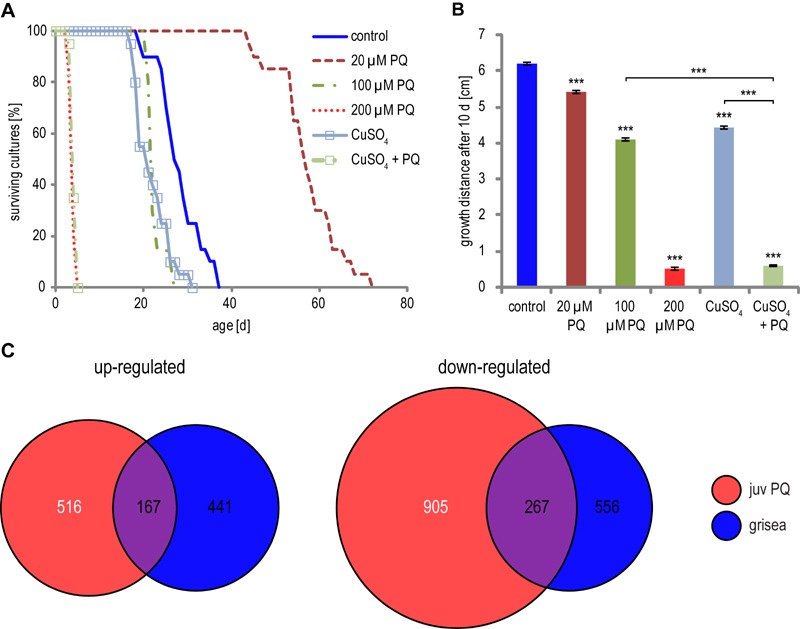
FIGURE 6: Effect of copper and PQ on lifespan and growth. **(A)** Lifespan of *P. anserina* on M2 medium supplemented with 20 µM PQ (n = 20; median lifespan = 56.5 days; p < 0.001), 100 µM PQ (n = 20; median lifespan = 21.6 days; p < 0.001), 200 µM PQ PQ (n = 20; median lifespan = 3.5 days; p < 0.001), 100 µM CuSO_4_ (n = 20; median lifespan = 21.5 days; p < 0.001) or 100 µM PQ and 100 µM CuSO_4_ combined (n = 3.8; median lifespan = 3 days; p < 0.001). P-values were determined in comparison with untreated control (n = 20; median lifespan = 27 days) by 2-tailed Wilcoxen rank sum test. **(B)** The growth distance of cultures from **(A)** was measured after 10 days on M2 medium. n = 20. P-Value against control or as indicated was determined by 2-tailed Wilcoxen rank sum test. **(C)** Differential transcript abundance of the wild type grown under PQ-stress and in the grisea mutant grown without additional PQ is depicted in Venn diagrams of up- (left) or down- (right) regulated transcripts (factor < 3; pV < 0.01) during PQ-stress in juvenile *P. anserina* and in the mutant grisea.

In previous investigations, it has been shown that excess copper has negative effects on *P. anserina *and that copper transport is tightly regulated 43. It is reasonable to assume that the addition of PQ influences the balance between the essential and the negative role of copper on the organism. To test this assumption we investigated the lifespan of *P. anserina* on medium supplemented with PQ and copper. Although the gene coding for high affinity copper transport is down-regulated during PQ-stress, copper can be transported into the cell by low affinity copper transport, if the copper concentration in the surrounding medium is high enough. To determine the lifespan under copper- and PQ-stress, mycelial pieces from cultures developed from ascospores after germination on sporulation medium were transferred to M2 medium supplemented with CuSO_4_ and PQ. The addition of low concentrations of PQ has a strong lifespan extending effect most likely reflecting a hormetic response 30. In contrast, addition of 100 µM CuSO_4_ or 100 µM PQ leads to decreased lifespans. Addition of one stressor alone results in a decrease of the median lifespan of 27 days (non-stressed cultures) to 21.5 days in copper supplemented medium and to 21.6 days in PQ containing medium (Figure 6A). Most strikingly, the addition of both, 100 µM PQ and 100 µM CuSO_4_ has a synergistic effect: cultures die very fast and have a median lifespan of 3.8 days. Consistent results were obtained for the effect of the two stressors on the growth rate. Strains challenged with either exogenous PQ or copper sulfate are characterized by reduced growth rates (Figure 6B). The simultaneous addition of copper sulfate and PQ to the medium leads to a complete growth arrest after one to two days, possibly caused by a synergistic effect of the two different stressors as the result of an increase of cytoplasmic copper beyond lethal thresholds.

Finally, we investigated the effect of copper depletion on global gene expression. We compared the differentially expressed genes (factor < 3; pV < 0.01) of the copper depletion mutant grisea 23 with the differentially expressed genes in juvenile PQ-stressed fungi and found 167 up-regulated and 267 down-regulated genes affected in both situations (Figure 6C). These numbers are much higher than statistically expected if regulations were random (Table 1). Moreover, statistical analysis revealed a higher similarity of transcript regulation in the grisea mutant and PQ-stressed juvenile wild type (odds ratio 6.29 / 8.41) than during aging and by PQ-treatement (odds ratio 3.19 / 3.21) (Table 1). We conclude that PQ-stress has a strong effect on copper homeostasis and therefore on copper-dependent gene expression.

### PQ-stress induced mitochondrial remodeling and the induction of programmed cell death

The current genome-wide transcriptome analysis revealed a strong effect of oxidative stress induced by PQ on gene expression. We observed a striking up-regulation of genes involved in mitochondrial remodeling which is expected, since PQ is active in ROS generation at the mitochondrial respiration chain. It appears that PQ-stress is indeed a good mimetic to induce mitochondrial ROS-stress as it naturally occurs during aging of biological systems. The effect of PQ-stress on cellular metal homeostasis verifies earlier conclusions of age-related increases in cytoplasmic copper levels. Such changes have been reported to occur during normal aging of the *P. anserina* wild type. The source of accumulating cytoplasmic copper has been suggested to be the mitochondrion 12,41,43 which is known to be a cellular copper store 47,48, but, although experimentally not addressed in *P. anserina *so far, copper release from the vacuole may also contribute to the age-related increase in cytoplasmic copper 49,50. A release of copper from mitochondria during aging of *P. anserina* appears to result from changes in mitochondrial membrane permeability 14. The age-related accumulation of cyclophilin D (PaCYPD), a component and regulator of the mitochondrial permeability transition pore, in the *P. anserina* wild type during aging 51 supports this conclusion and identifies a specific type of membrane permeabilization (for a review: 52). Previous work demonstrating that the over-expression of *PaCypD,* coding for cyclophilin D, accelerates aging via the induction of programmed cell death is further support of such a mechanism which is linked to pronounced mitochondrial membrane remodeling and the rupture of the outer mitochondrial membrane 21,53,54. The processes identified in the microbial model system *P. anserina* can explain the increase in cytoplasmic copper and the effect on copper-regulated gene expression as it has been described to occur during replicative senescence of human fibroblasts 43. They thus may be part of mechanisms involved in the control of programmed cell death and aging which are conserved from yeast to humans 43,55.

## MATERIALS AND METHODS

### Determination of hydrogen peroxide release

Measurement of hydrogen peroxide release was performed according to 56. After germination for 2 days at 27 °C in the dark on germination medium (standard cornmeal agar supplemented with 60 mM ammonium acetate 9, *P. anserina* was grown for 11 days on M2 medium (senescent) or used directly (juvenile). A piece from the growth front was transferred to a M2 plate (M2 medium: 0.25 g/L KH_2_PO_4_, 0.3 g/L K_2_HPO_4_, 0.25 g/L MgSO_4_ × 7 H_2_O, 0.5 g/L urea and 10 g/L yellow dextrin. Addition of 2.5 mg/L biotin, 50 mg/L thiamine, 5 mg/L citric acid × 1 H_2_O, 5 mg/L ZnSO_4_ × 7 H_2_O, 1 mg/L Fe(NH_4_)_2_(SO_4_)_2_ × 6 H_2_O, 2.5 mg/L CuSO_4_ × 5 H_2_O, 25 mg/L MnSO_4_ × 1 H_2_O, 50 mg/L Na_2_MoO_4_ × 2 H_2_O and 50 mg/L H_3_BO_3_ (after sterilization of the basal medium) supplemented with 0, 10 or 20 µM paraquat (PQ, Sigma-Aldrich, 856177) and grown for 4 days at 27°C and permanent light. Plates were flooded with a solution containing 2.5 mM 3,3-diaminobenzidine (DAB, Sigma-Aldrich), 0.02 mg/ml horseradish peroxidase (Sigma) and 100 mM Tris, pH 6.9 and incubated 3 h in the dark at 27°C. The staining solution was removed and the plate photographed.

Quantitative measurement of hydrogen peroxide was performed as described 7. For this measurement, *P. anserina *was cultivated in a similar manner as before, but on M2 medium instead of PASM 57 and to a final age of 6 d, 11 days and 15 d.

### Cloning of *P. anserina* *HyPer*-strains

The generation of *P. anserina* *HyPer* strains was performed by two fragment ligation. The fragment containing the *HyPer* gene was amplified using oligonucleotides HyPer-fwd (5’- CATTCGTTGGGGGATCCACC-3‘, Eurofins MWG Operon, Ebersberg, Germany) with restriction site BamHI and HyPer-rws (5’-CGTCTAGATTAAACCGCCTGTTTAA-3’, Eurofins MWG Operon) with restriction site XbaI and pHyPer-dMito (Evrogen) as template. The amplified product and the vector pExMtterhph 8 were digested with BamHI (Thermo Scientific, ER0051) and XbaI (Thermo Scientific, ER0681). The plasmid was ligated and used to transform *P. anserina* wild-type spheroplasts as described 58,59. Transformants were selected on hygromycin B supplemented medium.

### Measurement of cytoplasmic *in vivo* H_2_O_2_-levels

*P. anserina* was germinated as previously described and grown for 4 days on M2 medium. Cylindrical pieces of agar containing 6 days old *P. anserina* cultures were punched out of the plate, transferred into a 96-well plate and surrounded with 60 µl H_2_O. The fluorescence emission of the mycelium after exitation at 488 ± 5 nm (oxidized HyPer) and 420 ± 5 nm (reduced HyPer) was measured in a three minute interval at 530 ± 20 nm (Tecan, Safire2). The relative H_2_O_2_ levels are the average of the quotient of emission at 530 ± 20 nm of oxidized (excitation at 488 ± 5 nm) and reduced (excitation at 420 ± 5 nm) HyPer of three replicates of the treated probes substracted by the average quotient of the untreated control.

### RNA isolation

For transcriptome analysis three monokaryotic *P. anserina* ascospores of the wild-type "s" strain were germinated 2 days in the dark at 27°C. These cultures were directly used or - in order to generate strains of older age - grown for 4 days (middle-aged) and 8 days (senescent) under permanent light at 27°C on solid PASM medium. Subsequently, pieces of mycelium from the growth front of the PASM plates or directly from the germination plate (for juvenile cultures) were transferred onto solid PASM medium overlaid with a cellophane sheet and grown for 2 days (juvenile cultures) or for 3 days (middle-aged and senescent cultures) to obtain sufficient amounts of mycelium. The mycelium was scraped off, transferred to Erlenmeyer flasks containing liquid complete medium (CM: 70 mM NH_4_Cl, 7.3 mM KH_2_PO_4_, 6.7 mM KCl, 2 mM MgSO_4_, 1% glucose, 0.2% tryptone, 0.2% yeast extract, 5 mM FeCl_2_, 3.5 mM ZnSO_4_, 6.2 mM MnCl_2_, pH 6.5) and incubated at 27°C in permanent light and shaking, according to 9. After 24 h, PQ was added to final concentration of 200 µM and probes where shaken for 24 h. This procedure results in cultures with a final age of 6 days (juvenile), 11 days (middle-aged) and 15 days (senescent). The lifespans of the three isolates on PASM were recorded to ensure that they have a similar aging behavior. All three isolates had a lifespan of 15 days. Total RNA was isolated using a CsCl density gradient as previously described 23.

### SuperSAGE analysis

SuperSAGE analysis was performed by GenXPro GmbH (Frankfurt, Germany). Sequence tag identification and annotation were carried out as described 23. The relative gene expression was calculated by comparison of tpm of PQ-stressed samples with transcriptome data of untreated cultures (control) 19. The following factors were calculated: juv PQ, tpm 6 days PQ / tpm 6 days control; ma PQ, tpm 6 days PQ / tpm 6 days control; sen PQ, tpm 15 days PQ / tpm 14 days control; aging: tpm 14 days control/ tpm 6 days.

### Gene ontology

Gene ontology analysis was performed as described 19.

### Isolation of total protein extracts

Mycelial pieces from freshly germinated ascospores were transferred to M2 plates for 4 days (ma) or 7 - 9 days (sen). Subsequently, a piece of mycelium from the growth front was spread on a fresh M2 plate, overlaid with cellophane and grown for 3 days. To obtain juvenile cultures, germinated fungi were directly spread on M2 plates overlaid with cellophane and grown for 2 days. All cultures were transferred into Erlenmeyer flasks containing liquid complete medium and grown for 2 days to generate enough material. PQ was added to the indicated samples to a final concentration of 200 µM, 24 h before isolation. All incubation steps were performed at 27°C and permanent light, except germination which was executed in the dark. Proteins were isolated by grinding the mycelium under liquid N_2_ as described 10.

### Determination of SOD activity

For SOD activity determination in protein extracts an SOD determination kit 19160 (Fluka Analytical) was used. 1 µg protein extract was used per sample. The kinetic of pigment formation was recorded continuously in a 96-well plate reader (Tecan, Safire2) at 27°C. SOD activity was calculated during linear reaction.

### Quantitative photometric measurement of catalase activity

*In vitro* measurement of H_2_O_2_ degradation was carried out as described 36. The Δ*PaCatB*-strain was used as a control 36. 100 µg of total protein extracts were used.

### Quantitative Real-Time PCR (qRT-PCR)

Total RNA of the *P. anserina *wild type (the three RNA samples isolated for Super-SAGE analysis) was DNase digested with RNA-Plant kit (Machery-Nagel). Reverse transcription of 1 µg of DNase free RNA was performed using iScript kit (BioRad). The cDNA was diluted to a concentration of 10 ng / µl and 20 ng were used per qRT-PCR reaction (IQ SybrGreen SuperMix, Biorad). Three technical replicates were performed for each sample. The primers summarized in Table S2 were used as indicated. The PCR efficiency was determined as described 60. The relative expression was normalized to the reference genes *PaPorin* and *PaRpl19* with the following formula:

Relative expression = (root(E(Porin)^CP(Porin)*E(Rpl19)^CP(Rpl19)))/(E(target gene)^CP(target gene)) with E: PCR-Efficiency and CP: crossing point.

### Determination of respiration

Juvenile fungi were cultivated as described above. Small pieces of mycelium (dry weight 2 to 9 mg) were transferred into a high resolution respirometer (Oxygraph2-k, OROBOROS) and oxygen consumption was measured in CM medium. SHAM (Salicylhydroxamic acid, Sigma-Aldrich) and KCN were added to a final concentration of 4 mM and 1 mM, respectively, to inhibit respiration via complex IV and PaAOX respectively and to determine the portion of AOX dependent respiration.

### Lifespan determination

Lifespan of isolates was determined on M2 medium as described 61. After germination of monokaryotic ascospores, a piece of mycelium was transferred to control M2 medium and M2 medium supplemented with 20 µM, 100 µM and 200 µM PQ, 100 µM CuSO_4_ and 100 µM PQ and 100 µM CuSO_4_ combined. *P. anserina *was grown at 27°C under permanent light.

### Statistical analysis of data

Calculation of expected value for common regulation were done with: E(x) = n*M/N, where n and M are the number of regulated genes and N is the total number of genes. Independence and odds ratio were calculated using Fisher’s exact test. All other statistical calculations were done with student’s t-test or Wilcoxen rank sum test, as indicated. Asterisks indicate p-values: * p %lt; 0.05, **p < 0.01, *** p < 0.001.

## SUPPLEMENTAL MATERIAL

Click here for supplemental data file.

All supplemental data for this article are also available online at http://microbialcell.com/researcharticles/effect-of-paraquat-induced-oxidative-stress-on-gene-expression-and-aging-of-the-filamentous-ascomycete-Podospora-anserina/.
