# Supplementary material for: Effect of paraquat-induced oxidative stress on gene expression and aging of the filamentous ascomycete Podospora anserina
Source: Microb Cell. 2014 Jun 22;1(7):225–40. doi: 10.15698/mic2014.07.155 (PMC5349155; doi:10.15698/mic2014.07.155)
Supplement: Supplementary file 1 [file mic-01-225-s01.pdf]

Supplemental Data

A

PaNo.	gene	juv PQ	ma PQ	sen PQ	aging	grisea
Pa_2_510	*PaPks1*	0.49	0.08	0.35	3.11	
Pa_5_11880		0.04	0.05	0.00	1.10	0.11
Pa_7_11610		0.29	0.06	0.07	1.72	0.30
Pa_5_1990		0.10	0.06	0.06	1.13	0.24

0.2 0 5

B

PaNo.	gene	juv PQ	ma PQ	sen PQ	aging	grisea
Pa_1_15010	*PaAl-1*	6.15	1.33	4.30	0.88	1.33
Pa_1_15240	*PaAl-2*	5.88	3.00	0.70	2.07	1.89
Pa_2_610	*PaAl-3*	2.04	1.05	0.60	1.31	0.23

C

PaNo.	homolog	e-value	juv PQ	ma PQ	sen PQ	aging	grisea
Pa_5_11970	*ScFre1* / *ScFre2*	9.0E-32, 8.0E-30	0.05	0.13	0.22	0.39	0.00
Pa_7_5660	*ScFre1* / *ScFre2*	9.0E-29, 2.0E-28	0.42	1.52	0.40	0.85	0.68
Pa_5_10230	*ScFre1* / *ScFre2*	1.0E-23, 3.0E-27	1.21				
Pa_1_19630	*ScFre1* / *ScFre2*	5.0E-22, 2.0E-23	1.54	0.50	0.85	2.06	0.44
Pa_1_16410	*ScFre1* / *ScFre2*	2.0E-21, 1.0E-17	0.11	2.80	1.25	0.04	0.21
Pa_1_19550	*ScFre1* / *ScFre2*	2.0E-16, 6.0E-12	1.95	0.81	1.46	0.48	2.57
Pa_6_4210	*ScFtr1*	2.00E-55	1.42	2.06	0.55	0.38	0.31
Pa_6_4220	*ScFet3*	1.00E-130	0.30	0.44	0.26	1.11	0.13
Pa_2_530	*ScFet3*	3.00E-78	0.42	0.22	0.09	7.40	0.08
Pa_6_2550	*ScFet3*	3.00E-56	0.12	0.10	0.28	1.01	1.01
Pa_5_1200	*ScFet3*	7.00E-47	4.40	1.98	15.64	0.43	0.55

D

PaNo.	homolog	e-value	juv PQ	ma PQ	sen PQ	aging	grisea
Pa_6_1720	*AfSreA*	2E-40	1.30	18.08	2.52	0.18	0.47
Pa_4_4430	*AfSidA*	1E-119	3.26	2.99	4.12	0.64	2.49
Pa_5_4760	*AfSidF*	1E-107	3.12	2.57	5.09	0.78	1.09
Pa_3_11200	*AfSidD*	0.0	3.41	2.04	2.32	1.44	1.52
Pa_4_4640	*AfSidD*	0.0	1.05	0.45	0.34	0.90	0.48
Pa_5_1070	*AfSidD*	5E-92	0.87	0.52	1.62	0.99	0.85
Pa_4_4440	*AfSidC*	0.0	1.66	2.11	1.84	1.25	3.06
Pa_4_4640	*AfSidC*	1E-121	1.05	0.45	0.34	0.90	0.48
Pa_2_7870	*AfSidC*	1E-119	0.14	0.01	0.11	0.92	0.02
Pa_7_120	*AfMirB*	6E-88	4.14	4.95	4.04	0.44	2.34
Pa_5_3540	*AfMirB*	1E-117	2.26	1.70	1.02	0.90	0.73
Pa_5_4490	*AfSit1*	0.0	1.92	3.54	0.65	1.69	0.78
Pa_7_6950	*AfMirC*	1E-151	1.96	4.74	2.48	0.20	1.60
Pa_3_1250	*AfEstB*	2E-38	1.06	0.99	0.86	0.95	10.93
Pa_2_8330	*AfEstB*	1E-37	1.18	4.96	0.29	0.91	0.39
Pa_4_1300	*AfEstB*	8E-30	0.39	0.12	0.24	2.58	1.22

Supplemental Figure 1: Transcriptome data of transcripts associated with (A) melanin synthesis, (B) carotenoid synthesis, (C) reductive iron assimilation and (D) siderophore mediated iron uptake. Relative expression was calculated by dividing tpm of juvenile PQ-stressed samples by tpm of juvenile control (juv PQ), middle-aged PQ-stressed samples by middle-aged control (ma PQ), senescent PQ-stressed samples by senescent control (sen PQ), senescent control by juvenile control (aging) and of grisea by wild-type control (grisea).

Supplemental Table 1: GO enrichment analysis of transcriptome data PQ-stressed senescent wild-type cultures of *P. anserina*.

up-regulated

	GO ID	p-value	C	S	Description
CC	GO:0044429	1.73E-10	118	581	mitochondrial part
	GO:0005740	1.22E-07	83	408	mitochondrial envelope
	GO:0005743	1.31E-06	57	261	mitochondrial inner membrane
	GO:0019866	3.61E-06	57	269	organelle inner membrane
	GO:0005739	4.14E-06	176	1121	mitochondrion
	GO:0005759	7.53E-06	47	212	mitochondrial matrix
	GO:0044455	9.33E-06	38	159	mitochondrial membrane part
	GO:0031966	1.26E-05	73	386	mitochondrial membrane
	GO:0031975	1.52E-04	101	617	envelope
	GO:0031967	1.93E-04	96	584	organelle envelope
	GO:0005758	1.48E-03	12	40	mitochondrial intermembrane space
	GO:0000313	1.52E-03	11	35	organellar ribosome
	GO:0005761	1.52E-03	11	35	mitochondrial ribosome
	GO:0045275	1.81E-03	5	9	respiratory chain complex III
	GO:0005750	1.81E-03	5	9	mitochondrial respiratory chain complex III
	GO:0005744	2.07E-03	6	13	mitochondrial inner membrane presequence translocase complex
	GO:0044420	2.29E-03	4	6	extracellular matrix part
	GO:0005581	2.29E-03	4	6	collagen
	GO:0031970	2.38E-03	14	53	organelle envelope lumen
	GO:0000314	3.27E-03	6	14	organellar small ribosomal subunit
	GO:0005763	3.27E-03	6	14	mitochondrial small ribosomal subunit
	GO:0005578	3.27E-03	5	10	proteinaceous extracellular matrix
	GO:0016021	3.76E-03	241	1796	integral to membrane
	GO:0030015	4.84E-03	4	7	CCR4-NOT core complex
	GO:0031304	7.11E-03	6	16	intrinsic to mitochondrial inner membrane
	GO:0031305	8.42E-03	5	12	integral to mitochondrial inner membrane
BP	GO:0007005	2.65E-07	56	243	mitochondrion organization
	GO:0070585	2.29E-05	19	59	protein localization to mitochondrion
	GO:0072655	2.29E-05	19	59	establishment of protein localization to mitochondrion
	GO:0006839	2.67E-05	29	113	mitochondrial transport
	GO:0006626	5.03E-05	18	57	protein targeting to mitochondrion
	GO:0030026	2.65E-04	7	13	cellular manganese ion homeostasis
	GO:0055071	2.65E-04	7	13	manganese ion homeostasis
	GO:0006783	4.72E-04	9	22	heme biosynthetic process
	GO:0006779	4.83E-04	11	31	porphyrin-containing compound biosynthetic process
	GO:0006778	6.59E-04	11	32	porphyrin-containing compound metabolic process
	GO:0042168	6.96E-04	9	23	heme metabolic process
	GO:0006627	8.43E-04	4	5	protein processing involved in protein targeting to mitochondrion
	GO:0055114	8.53E-04	66	388	oxidation-reduction process
	GO:0033014	1.53E-03	11	35	tetrapyrrole biosynthetic process
	GO:0045333	1.72E-03	40	215	cellular respiration
	GO:0033013	1.98E-03	11	36	tetrapyrrole metabolic process
	GO:0055085	2.20E-03	111	740	transmembrane transport
	GO:0030150	2.90E-03	7	18	protein import into mitochondrial matrix
	GO:0000002	2.95E-03	12	43	mitochondrial genome maintenance
	GO:0000963	3.29E-03	5	10	mitochondrial RNA processing
	GO:0032543	4.30E-03	15	62	mitochondrial translation
	GO:0046148	4.30E-03	15	62	pigment biosynthetic process
	GO:0010508	4.47E-03	8	24	positive regulation of autophagy
	GO:0007006	4.47E-03	9	29	mitochondrial membrane organization
	GO:0006673	4.86E-03	4	7	inositolphosphoceramide metabolic process
	GO:0006675	4.86E-03	4	7	mannosyl-inositol phosphorylceramide metabolic process
	GO:0017038	5.50E-03	24	120	protein import
	GO:0006091	5.58E-03	56	344	generation of precursor metabolites and energy
	GO:0042180	6.75E-03	113	781	cellular ketone metabolic process
	GO:0015980	7.19E-03	44	261	energy derivation by oxidation of organic compounds
	GO:0046364	7.75E-03	12	48	monosaccharide biosynthetic process
	GO:0009060	8.04E-03	23	117	aerobic respiration
	GO:0000050	8.83E-03	4	8	urea cycle
	GO:0006094	8.85E-03	11	43	gluconeogenesis
	GO:0051188	9.36E-03	29	159	cofactor biosynthetic process
	GO:0010506	9.83E-03	8	27	regulation of autophagy
MF	GO:0016679	2.47E-04	14	42	oxidoreductase activity, acting on diphenols and related substances…

	GO ID	p-value	C	S	Description
	GO:0015450	3.88E-04	9	21	P-P-bond-hydrolysis-driven protein transmembrane transporter activity
	GO:0045551	4.09E-04	6	10	cinnamyl-alcohol dehydrogenase activity
	GO:0008320	8.64E-04	9	23	protein transmembrane transporter activity
	GO:0022884	8.64E-04	9	23	macromolecule transmembrane transporter activity
	GO:0003824	1.38E-03	705	5566	catalytic activity
	GO:0004605	1.74E-03	3	3	phosphatidate cytidylyltransferase activity
	GO:0016783	2.07E-03	5	9	sulfurtransferase activity
	GO:0008121	2.42E-03	6	13	ubiquinol-cytochrome-c reductase activity
	GO:0016681	2.42E-03	6	13	oxidoreductase activity, acting on diphenols and related substances as…
	GO:0016746	2.54E-03	60	350	transferase activity, transferring acyl groups
	GO:0016747	5.00E-03	51	297	transferase activity, transferring acyl groups other than amino-acyl groups
	GO:0016491	5.51E-03	263	1920	oxidoreductase activity
	GO:0016782	5.72E-03	6	15	transferase activity, transferring sulfur-containing groups
	GO:0043115	6.33E-03	3	4	precorrin-2 dehydrogenase activity
	GO:0004325	6.33E-03	3	4	ferrochelatase activity
	GO:0016410	6.81E-03	25	125	N-acyltransferase activity
	GO:0008080	9.22E-03	23	115	N-acetyltransferase activity
	GO:0004479	9.56E-03	5	12	methionyl-tRNA formyltransferase activity

down-regulated

	GO ID	p-value	C	S	Description
BP	GO:0006260	5.57E-04	69	275	DNA replication
	GO:0006083	1.61E-03	8	15	acetate metabolic process
	GO:0030989	3.87E-03	4	5	dynein-driven meiotic oscillatory nuclear movement
	GO:0051691	4.40E-03	8	17	cellular oligosaccharide metabolic process
	GO:0019236	4.67E-03	29	104	response to pheromone
	GO:0043406	5.19E-03	3	3	positive regulation of MAP kinase activity
	GO:0000169	5.19E-03	3	3	activation of MAPK activity involved in osmosensory signaling pathway
	GO:0000187	5.19E-03	3	3	activation of MAPK activity
	GO:0000189	5.19E-03	3	3	MAPK import into nucleus
	GO:0000208	5.19E-03	3	3	MAPK import into nucleus involved in osmosensory signaling pathway
	GO:0032005	5.20E-03	12	32	signal transduction involved in conjugation with cellular fusion
	GO:0007124	5.47E-03	25	87	pseudohyphal growth
	GO:0010033	5.86E-03	45	181	response to organic substance
	GO:0032787	6.57E-03	56	236	monocarboxylic acid metabolic process
	GO:0019748	6.77E-03	15	45	secondary metabolic process
	GO:0036267	9.50E-03	22	77	invasive filamentous growth
	GO:0001403	9.50E-03	22	77	invasive growth in response to glucose limitation
MF	GO:0004497	1.65E-06	85	298	monooxygenase activity
	GO:0004339	1.40E-04	9	14	glucan 1,4-alpha-glucosidase activity
	GO:0016712	2.03E-04	19	47	oxidoreductase activity, acting on paired donors, with incorporation…
	GO:0016709	5.32E-04	35	115	oxidoreductase activity, acting on paired donors, with incorporation…
	GO:0008470	8.79E-04	5	6	isovaleryl-CoA dehydrogenase activity
	GO:0005337	9.04E-04	7	11	nucleoside transmembrane transporter activity
	GO:0050839	9.04E-04	7	11	cell adhesion molecule binding
	GO:0070330	9.06E-04	15	37	aromatase activity
	GO:0004312	9.60E-04	21	60	fatty acid synthase activity
	GO:0004085	9.74E-04	4	4	butyryl-CoA dehydrogenase activity
	GO:0052890	9.74E-04	4	4	oxidoreductase activity, acting on the CH-CH group of donors, with a flavin…
	GO:0018664	1.13E-03	12	27	benzoate 4-monooxygenase activity
	GO:0004316	1.36E-03	13	31	3-oxoacyl-[acyl-carrier-protein] reductase (NADPH) activity
	GO:0016872	1.84E-03	7	12	intramolecular lyase activity
	GO:0016491	2.30E-03	381	1920	oxidoreductase activity
	GO:0016229	4.03E-03	16	46	steroid dehydrogenase activity
	GO:0004474	4.18E-03	4	5	malate synthase activity
	GO:0005488	4.87E-03	1066	5839	binding
	GO:0016627	4.90E-03	44	171	oxidoreductase activity, acting on the CH-CH group of donors
	GO:0016705	5.33E-03	99	442	oxidoreductase activity, acting on paired donors, with incorporation…
	GO:0004708	5.52E-03	3	3	MAP kinase kinase activity
	GO:0047616	5.52E-03	3	3	acyl-CoA dehydrogenase (NADP+) activity
	GO:0015926	5.68E-03	20	64	glucosidase activity
	GO:0016405	5.74E-03	7	14	CoA-ligase activity
	GO:0033764	6.53E-03	15	44	steroid dehydrogenase activity, acting on the CH-OH group of donors…
	GO:0003995	8.05E-03	16	49	acyl-CoA dehydrogenase activity

All transcripts with differential expression (Factor < 3; pV < 0.01) in senescent PQ-stressed samples were analyzed. Entries high-lighted in red refer to terms discussed in the text. GO terms with pV < 1E-3 are shown. The GO terms referred to in the text are high-lighted in red. CC: Cellular Component; BP: Biological Process; C: Count (number of genes of respective GO term in the group (up- or down-regulated)); S: Size (total number of *P. anserina* genes with the respective GO term).

Supplemental table 2: Summary of primer sequences used for qRT-PCR.

PaNo.	gene	Primer	sequence
Pa_1_16400	*PaCtr1*	Pa_1_16400-1	CTCACAATGTCACACTCC
		Pa_1_16400-2	TTAGCGATCAGTGCTACC
Pa_2_4660	*PaSod2*	PaSod2-1	TGGCTGCTGTTGAGAAGACC
		PaSod2-2	TCGGCCGTCTTCCAGTTGAT
Pa_2_9780	*PaPorin*	Porin-RT-for	TCTCCTCCGGCAGCCTTG
		Porin-RT-rev	GAGGGTGTCGGCAAGTTC
Pa_3_1710	*PaAox*	PaAox1-1	CGCTGACAGAAGCTCAAT
		PaAox1-2	ATTCGTGGCGAGATCAAG
Pa_3_10440	*PaCtr3*	PaCtr3-1	GGCGATGTACTACAATGG
		PaCtr3-2	CCGTCGTCGTAATACTTC
Pa_4_650	*PaRpl19*	PaRpl19-1	AGATCTCCAACGCCAACTCC
		PaRpl19-2	TCGTGGTAGAGGTGCTTGTC
Pa_4_4770	*PaCtr2*	Pa_4_4770-1	CTGACGAGCACATACATGG
		Pa_4_4770-2	CGACGTTCATCGTCATCAC
